# Editorial: Morphological changes in immune cells for precision sepsis treatment

**DOI:** 10.3389/fphar.2026.1830509

**Published:** 2026-03-24

**Authors:** Yawen Xie, Na Cui

**Affiliations:** Department of Critical Care Medicine, Beijing Chao-Yang Hospital, Capital Medical University, Beijing, China

**Keywords:** cytokine, immune, immunosuppression, inflammation, macrophage, sepsis

## 1 Introduction

Sepsis, a life-threatening organ dysfunction caused by a dysregulated host response to infection, remains a major critical care challenge with persistently high global morbidity and mortality (Global Sepsis Collaborators, 2025). Immune dysregulation is the primary driver of sepsis progression. As the central executors of immune response, immune cells undergo dynamic morphological changes in transcriptional profiles, subset composition, functional phenotypes, which directly reflect the immune status. Early identification and real-time monitoring of these changes are vital for precise subtyping, optimized therapies, and improved outcomes (Cajander et al., 2024). However, traditional immune assessments fail to capture cellular heterogeneity, while complex multi-omics analyses lack clinical translatability. This Research Topic, including six original studies and one review, explores immune cell morphological changes in sepsis from transcriptional, subset, and functional perspectives, offering new evidence for early diagnosis and immunotherapeutic targets.

## 2 Transcriptional morphological features: core immune targets

The transcriptome underpins the morphology and function of immune cells. By integrating high-throughput sequencing with machine learning, studies have identified key sepsis-related immune regulatory genes. Yang et al. analyzed 1,166 samples, developing a 28-gene signature (AUC = 0.970) distinguishing sepsis from health controls and a 13-gene signature (AUC = 1.000) differentiating sepsis from SIRS. *PRKACB*, a crucial myeloid-associated hub gene downregulated in sepsis, is primarily expressed in macrophages. Reduced expression of PRKACB enhances TNF-α/IL-1β release and impairs the viability of macrophages, making it a target for early diagnosis and immunotherapy.

Furthermore, Cheng et al. identified three critical transcriptional biomarkers (*BMX, GRB10*, and *GADD45A*) with robust diagnostic performance. These hub genes were associated with immune response, as evidenced by the significant correlation with infiltrating immune cells. These authors not only delineated potential regulatory mechanisms via mRNA-miRNA-lncRNA interaction networks, but also predicted candidate drugs targeting the above three biomarkers. The above breakthroughs pinpointed the promising diagnostic and therapeutic significance of immunomorphological biomarkers in transcriptional level.

## 3 Cellular subset morphological features: immune heterogeneity and subtyping

The heterogeneity of immune cells contributes to different responses to sepsis treatment. Pi et al. revealed reduced regulatory interactions between naive Treg and PD1^+^CD152^+^TIGIT^+^ effector memory CD4^+^T cells in sepsis through mass cytometry. A novel subset signature featured by expanded CD45RA^−^CX3CR1^+^CTLA4^+^CD4^+^ T cells, CD45RA^-^17A^+^CD4^+^ T cells, CD15^+^CD14^+^ monocytes, and Ki67^+^ B cells, yielded AUC = 0.90 (discovery) and 0.94 (validation), thereby enabling precise immunophenotypic subtyping.

Similarly, Zhang et al. developed a risk model for early prediction of intra-abdominal candidiasis (IAC) in elderly sepsis patients, identifying reduced total T cells, CD28^+^CD8+T cells, and CD38^+^CD8+T cells as independent risk factors alongside clinical parameters like gastrointestinal perforation and renal replacement therapy. CD28 downregulation impairs T cell function, while CD38^+^CD8+T cell expansion indicates exhaustion. The machine learning-based model (AUC = 0.840 training, 0.783 test) outperforms the *Candida* score and facilitates IAC risk stratification in elderly sepsis patients.

## 4 Functional morphological features: linking phenotypes to treatment

To further investigate the underlying mechanisms, the relationship between morphological changes and cellular function was analyzed. The cytokine profile is a critical indicator of immune cell function. Yang et al. demonstrated that decreased PRKACB expression not only enhanced the release of proinflammatory cytokines (TNF-α and IL-1β), but also compromised the viability of macrophage in sepsis. A similar cytokine signature was discovered by Hernández-Jiménez et al. as a potential predictor of positive blood cultures in sepsis patients. They confirmed the relationship between positive blood cultures and cytokine profiling (reduced TNF-α, IL-1β, IL-6, IL-8, and increased IL-10) following *ex vivo* LPS challenge, positioning TNF-α production capacity as an effective diagnostic marker (AUC = 0.83).

Cellular metabolism is another relatively indirect manifestation of immune function. Watanabe et al. revealed that improved resistance to *Listeria* infection was associated with the metabolism reprogramming of CD11b^+^Ly6C^high^ myeloid cells in sepsis-surviving mice. Multi-omics analysis further focused on the lipid metabolism and indicated the association between lipid accumulation and elevated IFN-γ levels in CD11b^+^myeloid cells. Yang et al. also highlights glycolytic shift and lipid accumulation in macrophage as immune dysregulation drivers of sepsis and targeting SDH/ACLY or HIF-1α/Nrf2/HO-1 shows promise. This metabolic rewiring presents a potential therapeutic target for bolstering immune defense during and after sepsis.

Furthermore, Yang et al. reviewed the molecular mechanisms underlying the sepsis-induced immune dysfunction and organ injury. In addition to cytokine profiles, programmed cell death of immune cell also directly influences immune competence and function. Apoptosis of CD4+T cell leads to T cell exhaustion and adaptive immunosuppression. Pyroptosis of macrophage and endothelial cell exacerbates the cytokine storm and aggravates hyperinflammation state. Besides, the immune-coagulation crossover represents an important indirect mechanism responsible for attacking solid organs in sepsis, such as NETs and immunothrombosis.

## 5 Cutting-edge immunotherapy and future directions

Advances in sepsis immunotherapy are increasingly anchored in immune cell morphological features. Above articles indicates the preclinical potential of the drugs targeted on morphological changes in immune cells, including immune checkpoint inhibitors (PD-1 inhibitors and CTLA4 inhibitors), core gene-targeted interventions (PRKACB expression and small-molecule inhibitors for BMX,GRB10, and GADD45A), immune subset-specific therapies (CD28 agonists enhancing T cell activation, anti-CD38 antibodies alleviating immunosuppression), and metabolic modulators (such as PPARγ agonists regulating macrophage lipid metabolism, SDH inhibitors suppressing pro-inflammatory responses). In the future, key priorities include constructing multi-center dynamic immunomorphological databases to track longitudinal changes and identify immune transition nodes, developing point-of-care detection devices for core genes (e.g., PRKACB), subsets (e.g., CD28^+^CD8+T cells), and cytokines (e.g., TNF-α) for real-time clinical monitoring, advancing mechanism research via animal models and organoids to validate regulatory pathways, conducting subtype-specific clinical trials to develop early diagnosis models and personalized therapeutic strategies.

Immune cell morphological changes are central to sepsis immune dysregulation and precision treatment (Giamarellos-Bourboulis et al., 2024). In summary, the articles compiled in the Research Topic provided a multifaceted immune-profiling of sepsis from the perspective of transcription, lymphocyte subtyping and cellular function. Several identified morphological properties of immune dysfunction displayed diagnostic performance of sepsis, such as the expression of hub genes, immunophenotype features of certain lymphocyte subtypes, cytokine profiles, and so on. With progress in bedside technologies and clinical trials, individualized strategies based on these changes may improve sepsis prognosis, propelling the development of precision medicine in sepsis.
